# Mobile Phone-Based Joint Angle Measurement for Functional Assessment and Rehabilitation of Proprioception

**DOI:** 10.1155/2015/328142

**Published:** 2015-10-25

**Authors:** Quentin Mourcou, Anthony Fleury, Bruno Diot, Céline Franco, Nicolas Vuillerme

**Affiliations:** ^1^Univ. Grenoble-Alpes, AGIM, La Tronche, France; ^2^Univ. Lille, 59000 Lille, France; ^3^Mines Douai, URIA, 59508 Douai, France; ^4^IDS, Montceau-les-Mines, France; ^5^Institut Universitaire de France, Paris, France; ^6^Laboratory for Ergonomics and Work-related Disorders, Center for Sensory-Motor Interaction (SMI), Department of Health Science and Technology, Univ. Aalborg, Denmark

## Abstract

Assessment of joint functional and proprioceptive abilities is essential for balance, posture, and motor control rehabilitation. Joint functional ability refers to the capacity of movement of the joint. It may be evaluated thereby measuring the joint range of motion (ROM). Proprioception can be defined as the perception of the position and of the movement of various body parts in space. Its role is essential in sensorimotor control for movement acuity, joint stability, coordination, and balance. Its clinical evaluation is commonly based on the assessment of the joint position sense (JPS). Both ROM and JPS measurements require estimating angles through goniometer, scoliometer, laser-pointer, and bubble or digital inclinometer. With the arrival of Smartphones, these costly clinical tools tend to be replaced. Beyond evaluation, maintaining and/or improving joint functional and proprioceptive abilities by training with physical therapy is important for long-term management. This review aims to report Smartphone applications used for measuring and improving functional and proprioceptive abilities. It identifies that Smartphone applications are reliable for clinical measurements and are mainly used to assess ROM and JPS. However, there is lack of studies on Smartphone applications which can be used in an autonomous way to provide physical therapy exercises at home.

## 1. Introduction

Joint movement and sensorimotor control can actually be assessed with range of motion and proprioception measurements. Range of motion (ROM), which is the measurement of the extent of a movement of a joint, is used to evaluate and classify joints impairments in patients or the efficacy of certain rehabilitation program. It could be performed in various ways such as, for example, a simple visual estimation or high speed cinematography, in passive or active condition. It is well recognized that proprioceptive function is crucially important for balance, posture, and motor control. Proprioception, which can be defined as the perception of the position and of the movement of various body parts in space, is generally composed by the two following modalities: joint position sense and the sensation of limb movement. On the one hand, “joint position sense” (JPS) is relative to the awareness of the position of the members or segments against each other [1]. On the other hand, “kinaesthesia” is defined as the sensation of the motion to locate the different parts of the body and to evaluate their movement (velocity and direction) and the static part is named statesthesia. Proprioceptive alterations resulting either from diseases, accidents, trauma, surgery, or normal ageing may lead to necessitating specific rehabilitation to prevent injuries and reduce balance deficits. Indeed, it has been shown that proprioception is more important than vision to maintain balance in elderly people population and a decrease in proprioception increases the risk of falling [2, 3]. Moreover, proprioceptive physical activities have previously been shown to improve balance control in elderly [4].

Clinicians and clinical researchers usually employ specific and dedicated methods and tools to measure and improve proprioceptive function. Effects of therapies and robot-based rehabilitation therapies on proprioceptive function are well explored [5], as the assessment of proprioception with all its testing methods [6]. To measure JPS, clinicians mainly use apparatus such as goniometers, inclinometers, or video in a controlled environment under the direction of a medical staff member. Recently, new measurement tools appear with the noteworthy particularity to be based on an unavoidable object of daily living: the Smartphone. Smartphones have led to significant improvements in healthcare systems [7, 8]. They have the advantage of being small, easy to use, affordable, and connected and of including an inertial motion unit (IMU) composed of 3D accelerometer, magnetometer, and gyroscope almost as standard (only one of gyroscopes or magnetometers can be absent). Interestingly, these built-in inertial sensors allow detecting and monitoring both linear and angular movements of the phone. To provide this range of motion, fusion algorithms can be used and some of them have already been validated in several studies with dedicated IMU systems [9]. Smartphones can then be used as goniometric tools [10]. However, Smartphones also contain additional standard technologies such as a screen display, an audio system, or a tactile feedback system for the interaction with the user of the device. In this context, it could be used independently to perform self-measurements during home-based training. At this point, however, to the best of our knowledge, no review has been published on validated Smartphone tool allowing self-measurement and/or providing physical therapy exercises which may be performed in an autonomous way. Along these lines, the present paper was designed to report Smartphone applications that are currently used for measuring and improving proprioceptive abilities.

The remaining of this paper is organized as follow.  Section 2 describes related works for the assessment of functional and proprioceptive function abilities using Smartphone-based system. Then, advantages of autonomous self-measurement and training are discussed in Section 3. Current and future works are presented in Section 4. Conclusions are finally drawn in Section 5.

## 2. Related Works on Smartphone-Based Systems

### 2.1. Clinical Assessment

Assessment and training of functional and proprioceptive abilities are based on a variety of tests for ROM, JPS, kinaesthesia, force sense, and balance [39]. Passive and active conditions can be used to, respectively, bias joint mechanoreceptors or stimulating joint and muscle-tendon mechanoreceptors [40].

Assessing ROM is used to quantify baseline limitations of motion. It has been demonstrated that ROM measures depend on the number of degrees of freedom of the joint, the initial position, the direction of the movement, and diurnal variation [41].

Assessing JPS consists in different exercises of joint position matching during which the patient is asked to get back to a specified angular position from a neutral one without using visual information. Different factors are likely to influence performances of such a test (e.g., time spent in the expected position [42]) and it has been shown that passive matching, from such range of motion measurement, is more difficult to measure reliably than active one [43]. Such a test is usually performed by a physiotherapist using goniometer to measure joint angle.

Studies about clinical assessment of proprioceptive function, mainly JPS, are briefly presented in Table 1 and described according to the following plan: (Section 2.2) spine proprioception assessment, (Section 2.3) upper extremity proprioception assessment, and (Section 2.4) lower extremity proprioception assessment.

### 2.2. Spine Proprioception Assessment

#### 2.2.1. Cervical Spine Proprioception Assessment

For the cervical range of motion (CROM), JPS or ROM testing is used to measure head repositioning accuracy which leads to great errors for people with neck disorders [44, 45]. A laser-pointer is mounted at the top of individual's head and a handled button can switch it on to mark head position before and after head rotation. Differences in the placement of the marks are measured in millimeters and can be calculated in degrees or directly recorded in degrees with certain devices [46].

Active and passive CROM can be measured with Smartphone. Two recent studies have assessed validity and reliability of Smartphone applications to measure CROM [11, 12].

The first one used two commercial applications on an iPhone 4 and an iPhone 3GS: Clinometer (Plaincode Software Solutions, Stephanskirchen, Germany) and Compass (Apple, Cupertino, USA) [11]. These apps were compared to a specific CROM gold standard device compound of eyeglasses with three inclinometers placed at three different positions: one near the left ear for flexion/extension (sagittal plan) and another for the lateral flexions on the forehead (frontal plane). Both are gravity dependent. The last positioning was the top of the head. For this one, the magnetic dependence was compensated by placing an adapted brace. Measures were taken with the Smartphone placed on left and right side of the head aligned with the ear or with the eyes depending of the observed motion. Head rotations were measured with the iPhone placed on individual's head with the arrow aligned with the nose. Each participant performed maximal neck movement for each rotation, flexion, and extension. Twenty-eight healthy volunteers (23 ± 6 years) were observed by two different groups of two students in physical therapy. Results were analyzed with intraclass correlation coefficient (ICC) for validity and reliability. For concurrent validity, ICC values were between 0.50 and 0.65 but <0.50 for rotation. For intraobserver reliability, ICC values were between 0.65 and 0.85. For interobserver reliability, ICC values were under 0.60. Authors concluded that Smartphones have good intrarater reliability but lower interrater reliability. Validity is good for movements in sagittal and frontal planes but poor for rotation. This scientific work validates the use of a Smartphone application for the active angle measurement of neck in the sagittal and frontal planes.

The second study used a customized Android application compared with a validated gold standard three-dimensional motion analysis system (VICON, UK) [12]. It recorded motion with three reflective markers tracked by VICON Nexus V1.7.1 and a 9-camera VICON MX motion analysis system. The phone was mounted on a helmet to capture head flexion, extension, and rotation. A magnetic yoke was placed around participant neck to compensate magnetic dependence. Twenty-one healthy participants were recruited and sixteen of them come for a second session. Participants were instructed to perform each movement with a manual guidance provided by the single examiner who determined the end of CROM. Results were analyzed with Spearman's correlation, ICC, and Bland and Altman plots (B&A) for validity. Reliability was assessed with ICC_(3,3)_, ordinary least products (OLP) regression, Standard Error of Measurement (SEM), Limit of Agreement (LOA), and Minimal Detectable Change (MDC). Validity results showed ICC values between 0.53 and 0.98, with a Spearman correlation coefficient ranged between 0.52 and 0.98. Intraobserver reliability was revealed by ICC values between 0.82 and 0.90 but under 0.33 for rotation. Authors established the validity and intrarater reliability for movements in sagittal and frontal planes but not rotation “likely due to magnetic field interference” [12]. This scientific work validates the use of a Smartphone application for the active angle measurement of neck in the sagittal and frontal planes.

#### 2.2.2. Lumbar Spine Proprioception Assessment

Investigation of the reliability and validity of Smartphone application for measuring spinal range of motion was explored in five studies [34–38].

Kolber et al. compared the use of the Smartphone application iHandy Level (iHandy Inc.) on an iPhone 4 (Apple, Cupertino, USA) and a gravity-based bubble inclinometer (model 12-1056, Fabrication Enterprises, White Plains, New York, USA) [34]. Thirty healthy participants were recruited and observed by two examiners. Five active types of spinal range of motion measurements were taken: thoracolumbopelvic flexion, isolated lumbar flexion, thoracolumbopelvic extension right lateral flexion, and left lateral flexion. Reliability was assessed using ICC_(3,*k*)_ for intrarater and ICC model 2 for interrater. Mean, SEM, and MDC were also calculated. Main results for validity presented ICC_(3,*k*)_ values > 0.86. ICC values for intra- and interobserver reliability are, respectively, greater than 0.80 and 0.81. Authors concluded that “the iHandy Level application on the iPhone is both reliable and comparable to bubble inclinometry” [34]. This scientific work validates the use of a Smartphone application for the active angle measurement of lumbar spinal range of motion.

Another routine clinical angle measurement is the angle of thorax rotation or rib hump, which is important for patients with scoliosis. Izatt et al. [35] and Franko et al. [36] evaluated a Smartphone application, Scoligauge (Ockendon Partners Ltd, UK), on an iPhone (Apple, Cupertino, USA) compared to the standardize scoliometer. For the first study [35], eight plaster torsos were used for measurements performed by nine examiners (four experienced spinal orthopedic surgeons, a specialist physiotherapist, an experienced spinal orthotist, two training grade registrars, and an inexperienced physiotherapist). Plaster torsos were placed on a standard bench during passive measurements. Intra- and interobserver variability were assessed by using mean absolute difference and 95% Confident Interval (CI) and ICC. Limit of Agreement (LOA) value was 6.2°. For intraobserver reliability, CI value was ±3.2° and ±4,9° for interobserver with an ICC value equal to 0.92. Authors concluded that “clinical judgements as a result of iPhone rib hump measurements can be made with confidence based on readings taken from the iPhone when combined with the acrylic sleeve” [35]. In the second study, sixty angles were randomly selected and measured by four orthopaedic medical providers [36]. Validity was confirmed using Pearson's correlation coefficient (CPP), whose result was equal to 0.99. Authors concluded that “the Scoligauge app is a convenient novel tool that replicates the function of a standard clinical scoliometer but with a potentially decreased financial cost and greater convenience for providers” [36]. These scientific works validate the use of a Smartphone application for the active angle measurement of thorax rotation.

Two more recent studies evaluated Scoligauge application (Ockendon Partners Ltd., UK) but in clinical case with patients [37, 38]. Balg et al. [37] recruited thirty-four patients with adolescent idiopathic scoliosis and measurements were made by two examiners, one spinal orthopedic surgeon and one physical therapy student. Statistical analysis uses ICC to assess inter- and intraobserver reliability and validity. Bland and Altman plots were also used. For validity, the ICC value was equal to 0.947 and mean difference is 0.4 degrees. Intra- and interobserver reliability were assessed with ICC values of 0.961 and 0.901 and mean differences of 0.0 and 0.1 degrees. Authors concluded that “this study proved that even without an adapter the Scoligauge iPhone application is valid and can be used in the clinical setting for scoliosis evaluation” [37]. Qiao et al. recruited sixty-four patients with adolescent idiopathic scoliosis, in which thirty-two patients had main thoracic scoliosis while the rest had main thoracolumbar/lumbar scoliosis [38]. Measurements were made by two spine surgeons. Cobb angles were measured from posteroanterior radiographs. Patients performed Adam's forward bend test. Each examiner performed two evaluations; retest was done after twenty minutes of interval. Statistical analysis used ICC. Intraobserver ICC values were 0.954 for scoliometer and 0.965 for Scoligauge. Interobserver ICC values were 0.943 for scoliometer and 0.964 for Scoligauge. Authors conclude that “Smartphone-aided measurement for ATR showed excellent reliability, and the reliability of measurement by either scoliometer or Scoliogauge was influenced by Cobb angle where reliability was better for curves with larger Cobb angles” [38]. These scientific works validates the clinical use of a Smartphone application for the active angle measurement of spinal range of motion with patient.

### 2.3. Upper Extremity Proprioception Assessment

#### 2.3.1. Shoulder Proprioception Assessment

Functional and proprioceptive abilities on extremities such as shoulder usually use universal goniometer to measure active or passive range of motion. However, laser-pointer devices can also be a solution for this specific joint [47]. Laser pointer can be used to measure, in millimeter, differences between joint movements from a position to another and then calculate joint position in degree like universal goniometer. JPS tests can evaluate the ability of the individuals to reproduce a specific movement and the precision to access a specific angle target. These tests must be conducted with full knowledge of their limitations. It can involve some cognitive component, and the size and speed of movement should be standardized [39]. Active and passive shoulder ROM can therefore be measured with Smartphone. Six studies can be listed with the aim of studying validity and reliability of Smartphone applications for shoulder ROM [13–17, 48]. These studies will be described in the next paragraphs.

Shin et al. used a previously cited commercial application, on a Samsung Galaxy S: Clinometer [13]. The application was compared with a standard double-arm goniometer. The Smartphone was attached on the wrist (ventral side of the forearm) with the help of an armband. Observed shoulder passive and active movements were forward flexion, abduction, external rotation with the arms at the sides, external rotation at 90 degrees abduction, and internal rotation at 90 degrees abduction. Forty-one volunteers were observed by two orthopedic resident doctors and one orthopedic surgeon. Reliability was evaluated with ICC_(2,1)_ for interobserver and ICC_(3,1)_ for intraobserver. Results were greater than 0.70 for intraobserver except for internal rotation at 90° abduction (0.63–0.68). Interobserver's results were greater than 0.90. Validity was evaluated with SEM, MDC, B&A, and PCC. LOA was between 10 and 40°, ICC > 0.72, and PCC between 0.79 and 0.97. Authors concluded that “Smartphone application is reliable compared to the double-arm goniometer, although the between-day reliability remains to be established” [13]. This validates the use of a standard commercial Smartphone application for the active and passive angle measurement of shoulder range of motion.

Werner et al. used the same commercial application, Clinometer, but with another device that is an iPhone (Apple, Cupertino, USA) [14]. Clinometer was compared with a visual estimation and a standard goniometer. Twenty-four healthy adults and fifteen symptomatic patients were recruited. Measurements were performed by 5 examiners (one sport fellowship-trained orthopedic surgeon, one orthopedic sports medicine fellow, one orthopedic resident physician, one orthopedic physician assistant, and one medical student). Passive abduction and forward flexion were measured in standing position. They also measured external rotation with the arm at the patient's side, external rotation with the arm abducted at 90 degrees, and internal rotation with the arm abducted at 90 degrees; all were measured with the patient supine on an examination table. Reliability was evaluated with an ICC_(2,1)_ and this ICC for each measurement modality was compared by use of ANOVA with a Tukey post hoc test. Validity was evaluated using ICC_(2,1)_, B&A, and SEM. For this validity, with healthy participants, ICC > 0.60 and SEM < 4.3°. For symptomatic patients, ICC > 0.80 and SEM < 0.1°. For interobserver reliability, with healthy participants, ICC > 0.60 and SEM < 10.1°. For symptomatic patients, ICC > 0.60 and SEM < 5.8°. Authors concluded that “Smartphone Clinometer has excellent agreement with a goniometer-based gold standard for measurement of shoulder ROM in both healthy subjects and symptomatic patients” [14]. This result validates the use of a standard commercial Smartphone application for a clinical active and passive angle measurement of shoulder range of motion with patients.

Mitchell et al. used two commercial applications, GetMyRom (Interactive Medical Productions, LLC, USA) and DrGoniometer (CDM S.r.L.), with an iPhone device [15]. GetMyRom can measure JPS thanks to orientation sensors while DrGoniometer is a photo-based application that calculates the angles from markers positioned after the measurement. Both applications were compared with a standard goniometer. Ninety-four healthy women were recruited and measurements were made by one novice examiner and one expert at two different moments. Participants were instructed to perform active shoulder external rotation. Reliability, for inter- and intrarater, and validity were evaluated using ICC. Main results were as follows: for validity, ICC is equal to 0.94, for intraobserver, ICC is equal to 0.94, and for interobserver ICC = 0.79. Authors concluded that “both applications were found to be reliable and comparable to SG” [15]. In addition, the author plebiscite the use of the application based on the camera because of its potential for saving images. This scientific work validates the use of two specific Smartphone applications, which used inertial sensors or camera, for the active angle measurement of shoulder range of motion.

Oïhénart et al. used a custom application called iShould (Instrumented Shoulder Test) with iPhone 4 or iPod Touch devices (Apple, Cupertino, USA) [16]. iShould computes kinematics range of angular velocity (RAV) score, which quantifies the shoulder movement based on angular velocities and *P* score, which is based on the power of shoulder movement, directly from inertial signal sensors. The application is compared with another 3D kinematics sensors composed by three miniature capacitive gyroscopes (Analog device, ADXRS 250, 400°/s) and three miniature accelerometers (Analog device, ADXL 210, 5 g). Smartphone and sensors are attached to the anterior part of the humerus, using an armband. Five participants were recruited and performed active anterior elevation and extension, abduction and adduction, and internal and external rotation of the shoulder. Validity is evaluated using mean difference of RAV score and *P* score. Mean difference was 1.09% for RAV and 0.60% for *P* score. Authors concluded that “the application offers then an interesting alternative to the existing system” [16]. This work validates the use of a specific Smartphone application for the active angle measurement of shoulder range of motion.

Johnson et al. used a custom Android mobile application that mimics goniometer with the help of magnetometer on the first generation of Motorola Droid. Their application was compared with a universal standard double-arm goniometer [17] or with a full-scale motion capture system [48]. The Smartphone was used to collect angle in the same manner as the standard goniometer. Four passive shoulder's abductions were simulated in both seated and supine orientation. Only one participant was recruited for this pilot study and three therapists managed the measurement. Statistical analysis was performed with ANOVA, PCC, concordance correlation coefficient (CCC), and scatter plot were used to assess agreements, and B&A plots were used to compare differences. For validity, mean differences in B&A plots were −1.7° (in seated position) and 1.4° (in supine position). Intra- and interobserver reliability were presented with CCC values of 0.992 and 0.989. Authors concluded that “this study demonstrates the validity of the Smartphone goniometer application utilizing a built-in 3-axis magnetometer sensor when compared with a previously proven and universal goniometer. The Smartphone magnetometer-based goniometer also demonstrates comparably high reliability in measuring passive shoulder abduction ROM in both the seated and supine positions” [17]. These scientific works allow validating the use of a specific Smartphone application for the passive angle measurement of shoulder range of motion.

#### 2.3.2. Elbow Proprioception Assessment

Ferriero et al. also used the “DrGoniometer” application for elbow angle measurement [18]. This application works on iPhone and it was compared to a small plastic universal goniometer. In this proof-of-concept study, one participant was recruited and seven examiners assess measurements. Twenty-eight pictures of elbows of healthy subjects were taken at different angles, and this complete protocol was repeated a second time after one week. ICC was used for intrarater and interrater reliability. Validity was interpreted with a LOA between +4.51° and −5.75°. Intra- and interobserver ICC values were 0.998 for both. Authors concluded that the application “is reliable for elbow joint goniometry” [18]. This scientific work validates the use of a specific Smartphone application, which used camera, for the passive angle measurement of elbow range of motion.

### 2.4. Lower Extremity Proprioception Assessment

#### 2.4.1. Knee Proprioception Assessment

Functional and proprioceptive abilities tests for knee extremity usually use universal goniometers, in clinical practice, to measure active or passive ROM and evaluate the ability of individuals to reproduce a specific movement and the precision to reach a specific angle. Active and passive knee range of motion can therefore be measured using Smartphones. Ten researches can be listed with the aim of studying validity and reliability of Smartphone applications for knee ROM [19–28]. The next paragraph will briefly describes these studies.

Ockendon and Gilbert created their own Smartphone application called “Knee Goniometer” which is now published on the Apple Inc. App Store. The application is installed on an iPhone 3GS (Apple, Cupertino, USA) and compared with a telescopic-armed goniometer (Lafayette Instrument, Lafayette, IN) [19]. Five healthy participants were recruited and measurement was performed by two experienced and independent examiners. Each participant executed three different passive knee flexions, which were measured twice, separated by a time interval, on both right and left legs. Statistical analysis included B&A plot, Scatter plots, standard deviation (SD) of the difference, and PCC. For validity, LOA was equal to 15.2° and PCC was equal to 0.947. Intra- and interobserver main results for PCC were, respectively, 0.982 and 0.994. Authors concluded that “the iPhone goniometer [is] a reliable tool for the measurement of subtle knee flexion in the clinic setting when compared with the current standard bedside technique” [19]. This validates the use of a specific Smartphone application for the passive angle measurement of knee range of motion.

The “Knee Goniometer” application was also used to measure level of agreement with a goniometer for the assessment of maximum active knee flexion by an inexperienced tester [20]. An iPhone 3GS (Apple, Cupertino, USA) was also compared to a telescopic-armed goniometer (Lafayette Instrument, Lafayette, IN). Ninety-six healthy participants were recruited and measurements were performed by a graduate sports therapist inexperienced examiners. Participants were asked to perform three full active knee flexion movements from full knee extension to maximum knee flexion. Statistical analysis was conducted with a PCC test, a two-tailed paired *t*-test, an ICC to evaluate intratester reliability, and finally B&A. The paired *t*-test indicated a significant difference in results but it was not considered clinically significant. Intraobserver reliability is interpreted by an ICC value of 0.894 and a PCC value of 0.795. Authors concluded that “The iGoniometer demonstrated acceptable test-retest reliability and criterion validity for an inexperienced tester with healthy participants. There was a statistically significant difference between the iGoniometer and long arm goniometer measurements but this was not considered to be a level of difference that would have a clinical impact” [20]. This work validates the use of a specific Smartphone application with inexperienced testers for the active angle measurement of knee range of motion.

Matthew Ockendon has published another Smartphone application, called “Simple goniometer,” which also mimics standard two-arm goniometer. However, this application has no specific interface for knee JPS unlike “Knee Goniometer.” This application was used on an iPhone 3GS (Apple, Cupertino, USA) to assess its validity and reproducibility for JPS knee test compared with a universal goniometer [21]. Thirty-six healthy participants were recruited and measurements were made by two registered physiotherapists experienced in using the universal goniometer. Participants were instructed to actively and gently lunge forward with their dominant leg and remember the angle. They are then asked to return to the original position and to start again to reproduce the target angle. This was performed three times. Statistical analysis for reliability was determined using confidence intervals and for validity using PCC, ICC_(3,*k*)_, and B&A plots. Validity is interpreted with LOA results of 13,1° and PCC ranged from 0.96 to 0.98. Intraobserver ICC was between 0.97 and 0.99. Authors concluded that “the scores obtained from the simple Goniometer app for iPhone showed that there were concurrent validity and reliability for knee joint angle as compared with the universal goniometer” [21]. This scientific work allows validating the use of a specific Smartphone application for the active angle measurement of knee range of motion.

The “Knee Goniometer” application was also used to assess its validity and reliability beside experienced and novice clinicians [22]. It was installed on an iPhone 4 (Apple, Cupertino, USA) and compared to the universal goniometer as a gold standard. Six healthy students were recruited and all goniometric measurements were performed by six independent examiners. Participants were placed into passive knee flexion, always with the right leg. Each examiner made one measurement with both universal goniometer and Smartphone to each participant during one session. Three sessions were made with a fifteen-minute break between each one. Statistical analysis included the calculation of concordance correlation coefficient (CCC), SEM, Scatter plots, and B&A plots. For validity, CCC for expert was 0.982, CCC for novice was 0.983, CCC for all was 0.991, and CPP was −0.51. SEM was under 2.7° for goniometer and under 1.4° for the Smartphone application. In intra-observer reliability, CCC values varied between 0.998 and 0.999 for expert (clinicians) and varied between 0.997 and 0.999 for novice (students). In interobserver reliability, CCC was greater than 0.996 for expert, 0.998 for novice, and 0.997 for both. Authors concluded that “this study established that both the universal goniometer and the Knee Goniomter application were reliable for measurement of knee flexion angles by experienced clinicians and final year physiotherapy students using standardized protocols” [22]. This scientific work encourages the use of a specific Smartphone application for the passive angle measurement of knee range of motion.

Rwakabayiza et al. also used the “Knee Goniometer” application made by Ockendon for iPhone (Apple, Cupertino, USA) [23]. Universal goniometer is still used as a gold standard. Twenty healthy participants were recruited and twenty patients in acute postoperative knee prosthesis phase also participate. Measurements were realized by one specialist in orthopedic surgery, one physiotherapist, and one assistant doctor. Active and passive flexion-extension knee amplitudes were measured with the Norkin and White technique three times by each examiner. ICC for intra- and interobserver reliability was calculated. Validity results were produced on six patients, for an ICC value of 0.54 on Smartphone application in active extension and an ICC value of 0.92 for Smartphone application in active flexion. These results were better than goniometer's. Intraobserver reliability revealed a mean in ICC of 0.85 for healthy participants and 0.98 for patients. Interobserver reliability revealed a mean in ICC of 0.12 for healthy participants and 0.24 for patients. These last two results were considered “bad” by authors and with the Smartphone application with the goniometer as well. This prompted them to change their protocol to avoid fatigue-related bias. Authors concluded that “this study shows that the “Knee Goniometer” Smartphone application can be used in clinical practice as well as the universal standard goniometer to measure the range of motion of the knee” [23]. It provides, in addition to others, validation on patients in postoperative phase. This scientific work allows validating the clinical use of a specific Smartphone application for the active and passive angle measurement of knee range of motion with patients.

“Clinometer” application was used not only in shoulder but also in knee ROM [24]. It was set up on an iPhone 4 (Apple, Cupertino, USA) and used in comparison with a hand-held bilevel inclinometer as a gold standard. Forty-one healthy students were recruited along with two examiners. JPS was measured using passive knee extension. Each examiner had measured this extension three times with both instruments twice, with a break of one day. Statistical analyses were performed using Mann-Whitney test, ICC_(3,1)_, SEM, and MDC. Intraobserver results were ICC > 0.76 for the inclinometer, ICC > 0.72 for Smartphone application, and MDC < 5°. Interobserver results were ICC > 0.64 for the inclinometer, ICC > 0.64 for Smartphone application, and MDC < 8°. Authors concluded that “the results obtained at the level of the knee joint have similar characteristics between [the] two tools” [24]. This scientific work allows validating the use of a standard commercial Smartphone application for the passive angle measurement of knee range of motion.

Another clinical research was performed by Jenny with the “Angle” (Smudge App) Smartphone application [25]. A navigation system (OrthoPilot, Aesculap, Tuttlingen, FRG) was used as a gold standard. Ten patients, operated for end-stage osteoarthritis by navigation assisted for total knee arthroplasty, participated to the study. The knee was passively positioned at four full extensions and at maximal flexion angle. For each set of measurements, six navigated and six Smartphone data sets were obtained. Statistical analyses were performed with paired Student's *t*-test, and Spearman's coefficient of correlation, Bland and Altman plots, and intra- and interobserver reproducibility were assessed using ICC. Validity results were as follows: LOA was equal to 27.4°, *t*-test was not significant, Spearman's coefficient of correlation is 0.99, and B&A had good coherence. Intra- and interobserver ICC were, respectively, 0.81 and 0.79. Authors concluded that “the Smartphone application used may be considered as precise and accurate” [25]. This scientific work allows validating the clinical use of a standard commercial Smartphone application for the passive angle measurement of knee range of motion with patients.

Ferriero et al. used the “DrGoniometer” Smartphone photo-based application [26]. They studied its reliability in comparison to a universal goniometer. For the first experiment set, one healthy participant was recruited with four examiners, two experts (physiotherapists) and two novices (first-year physiotherapy students). Passive knee angle flexions were produced by an isokinetic device with the right leg fixed. Each examiner took twenty-five pictures at twenty-degree and eighty-degree knee flexion. The second set of experiments was made with ten healthy individuals assessed by ten examiners. Thirty-five pictures were taken at different knee angle measurements. This set was repeated one week later to evaluate inter- and intrarater correlation. Statistical analysis was carried out using ICC_(3,1)_ for intra- and interrater correlation, and Bland and Altman plot was used to evaluate differences. Resulting LOA ranged from −7.5° to +10.71°. Intra- and interobserver ICC were, respectively, 0.958 and 0.994. Authors concluded that “DrG is a reliable method for knee joint angle measurement […] the images of the measurement can be included in the patient's medical record as evidence of the quality of the care provided” [26]. This scientific work allows validating the use of a specific Smartphone application which used camera for the passive angle measurement of knee range of motion with patients.

For knee range of motion measurement, two types of Smartphone applications were used: sensor-based application and photo-based application [27]. Jenny et al. compared those two methods using Goniometer Pro (5fuf5) as the sensor-based application, DrGoniometer as the photo-based application, and a navigation system (OrthoPilot, Aesculap, Tuttlingen, FRG) as gold standard. Ten consecutive patients with end-stage osteoarthritis were selected and measurements were made by one examiner. Five measurements were obtained using each application. Statistical analysis was made using ANOVA test, paired difference, Level's test, Wilcoxon's test, Kendall's test, Spearman's test, and Bland and Altman plots. Results led to strong correlation and a good coherence. Authors conclude that “the camera smartphone application used in this study is fit for the purpose of measurement of the knee range of motion in a routine clinical setting and is substantially superior to inclinometer-based measurement” [27]. This scientific work validates the clinical use of a two specific Smartphone applications that used sensors or camera for the passive angle measurement of knee range of motion with patients. It aims to compare these applications and concluded that camera is superior to the sensors-based application. However, it must be noted that photo-based applications are not suitable for self-measurement.

Smartphone application for measuring range of motion in clinical practice was recently extended to new type of measure such as anterior tibial translation in anterior-cruciate ligament (ACL) deficient knees [28]. A specific Smartphone application, running on both Android and iOS, called “SmartJoint” was developed. This study compared this application, installed on both systems, with the arthrometer KT 1000 (Med Demetric, Kentucky, USA). Thirty-five patients with chronic ACL-deficient knees scheduled for ACL reconstruction were selected. Measurements were performed by two independent examiners. The Lachman test was performed three times on each knee with all devices. Statistical analysis used ICC to compare intertest, intraobserver, and interobserver reliability. Results were a mean ICC of 0.797 for uninvolved knee and mean ICC of 0.987 for involved knee; mean ICC of 0.973 for uninvolved knee and mean ICC of 0.989 for involved knee; and mean ICC of 0.957 for uninvolved knee and mean ICC of 0.992 for involved knee, respectively. Authors conclude that “the performance of SmartJoint is comparable and highly correlated with measurements obtained from KT 1000” [28]. This validates the clinical use of a specific Smartphone application for the passive angle measurement in Lachman's test.

#### 2.4.2. Hip Proprioception Assessment

Detecting abnormal Femoral Neck Anteversion (FNA) is important for physiotherapist to identify lower limb problems. Measuring FNA is possible with the angle formed by the vertical line and the tibial crest, when the greater trochanter is most prominent laterally. Yoon et al. compared the reliability of the method to measure FNA, including the comparison between an industrial digital inclinometer (GemRed DBB, Gain Express Holdings, Ltd., Hong Kong, China) as gold standard and an iPhone (Apple, Cupertino, USA) with TiltMeter (IntegraSoftHN) application [29]. Nineteen hips were examined in ten healthy subjects observed by two physical therapists. Three sessions of each method were repeated with one hour between sessions. Statistical analysis used ICC, SEM, PCC, and Kolmogorov-Smirnov *Z* test. Intraobserver ICC_(2,3)_ is 0.95, and SEM ranged from 1.9° to 2.2°. Interobserver ICC_(2,3)_ is 0.85, and SEM is equal to 4.1°. Authors concluded that “using a Smartphone with an inclinometer application during the TCAT showed comparable reliability to a digital inclinometer” [29]. This scientific work validates the use of a standard commercial Smartphone application for the passive angle measurement of hip proprioception.

Smartphone applications could also be used in addition to or instead of conventional techniques and computer-assisted surgery. Peters et al. try to improve acetabular cup orientation in total hip arthroplasty by using Smartphone technology [30]. They used two applications, Angle (Smudge Apps) and Camera Protractor Lite (YJ Soft) on an iPhone (Apple, Cupertino, USA). Angle application directly measures angle with the help of accelerometer while Camera Protractor Lite displays a protractor through the phone camera. Standard postoperative pelvic X-rays are used a gold standard. Fifty patients who need primary total hip arthroplasty operations were selected. Measurement was realized by a surgeon and their first assistant. The Angle application was used for the inclination of the acetabular cup and the Camera Protractor application was used to determine anteversion. Statistical analysis compared differences between intraoperative and postoperative angles. Results showed that differences were less than 5% between before and after operation. Authors concluded that “the use of the iPhone for acetabular cup placements is quick and accurate” [30]. This encourages the clinical use of two standard commercial Smartphone applications for hip angle measurement in surgery.

Reliability and concurrent validity of a Smartphone application to measure hip joint range of motion were assessed by Charlton et al. [31]. Measurements obtained with a custom Smartphone application, called “Hip ROM Tester,” were compared with those obtained with a camera marker-based 3DMA system (Vicon, Oxford, UK) and with a bubble inclinometer. Twenty healthy participants were recruited and all tests were conducted by one physiotherapist. These tests were passively performed by movements of flexion, abduction, adduction, supine internal and external rotation, and sitting internal and external rotation. Intratester reliability was performed using ICC, CV, and SEM values. Validity is performed using means, standard deviation, and ICC. Validity tests resulted in ICC_(2,3)_ > 0.88 for 6 movements and ICC_(2,3)_ was equal to 0.71 for supine external rotation. Intraobserver tests resulted in ICC_(2,3)_ > 0.84 for 4 movements and ICC_(2,3)_ was between 0.63 and 0.68 for 3 movements. Authors concluded that “a Smartphone application provides a reliable and valid method of assessing passive hip joint ROM in young active males” [31]. This scientific work allows validating the use of a specific Smartphone application for the passive angle measurement of hip range of motion.

#### 2.4.3. Ankle Proprioception Assessment

Ankle ROM using Smartphone applications was studied in two studies [32, 33]. The first one compares the iHandy Level app (iHandy Inc.) on an iPhone (Apple, Cupertino, USA) with a digital, medically rated inclinometer (Baseline, Fabrication Enterprises Incorporated, USA) [32]. Twenty participants were recruited and measurements were made by two physiotherapy honor students in their final year of study. The test measures the ankle dorsiflexion range; participants were instructed to lunge forward, bringing their knee in contact with a vertical tape on the wall. Three measurements were performed and mean was used to perform analysis. ICC and CI were used for intra- and interrater reliability; SEM and Bland and Altman plots were also produced. Validity was evaluated using Pearson's product-moment correlation coefficients and resulted in 0.99. Intra- and interobserver ICC were, respectively, equal to 0.97 and 0.76. Authors concluded that “a smartphone with the iHandy Level app can measure ankle dorsiflexion with high reliability as well as construct and criterion validity” [32]. This work validates the use of a specific Smartphone application for the active angle measurement of ankle dorsiflexion.

The second study [33] evaluated a Smartphone application, Tiltmeter (IntegraSoftHN, Carlos E. Hernández Pérez) on iPhone 4 and 4S (Apple, Cupertino, USA) during the weight bearing lunge test. A digital inclinometer (Laser Depot, Adelaide, Australia) was used as a gold standard. Twenty healthy participants were recruited and measurements were performed by two podiatrists. Examiners helped participants to slowly move the right foot back until they were able to hold the lunge position with the heel on the floor and with the right foot straight and perpendicular to the wall. Intrarater reliability was determined using ICC_(2,1)_ and 95% CI. Interrater reliability was determined using ICC_(2,2)_ and 95% CI. Validity between both devices was explored using Bland and Altman plots and ICC and resulted in a mean value of 0.83. Intra- and interobserver ICC were ranged between 0.81 to 0.85 and 0.80 to 0.96. Authors concluded that “the use of the TiltMeter app on the iPhone is a reliable measure of ankle range of motion in healthy adults” [33]. This scientific work validates the use of a commercial standard Smartphone application for the active angle measurement for weight bearing lunge test.

## 3. Using Smartphone for Proprioception Rehabilitation in Autonomous Way

All the previously presented studies assess the use of the Smartphone for functional and proprioceptive abilities assessment. Most of them only focus on joint angle measurements through ROM. They all conclude that Smartphone applications, which are sensor-based or camera-based, are reliable and valid for measuring angle compared to some gold standard as goniometer, bubble inclinometer, 3D navigation system, or even scoliometer for assessing JPS. However, some limitations are pointed out by authors. In cervical range of motion, both studies concluded that rotation evaluations are not reliable due to magnetic field interference. Gimbal lock effect may also decrease reliability for JPS if its effect is not taken into account in the measurement protocol. ISB recommendation proposes, for each joint, a standard for the local axis system in each articulating segment or bone and thus can bring solutions to avoid Gimbal lock effect in protocols [49, 50]. In their recent review of Smartphone goniometric tools, Milani et al. [10] concluded that there are no validation studies focusing on Smartphone application in dynamic conditions. We fully agree with this conclusion. We further state that while the Smartphone is now validated as a reliable measurement tool and can be used in clinical practice, there are no studies which use the power of the Smartphone as both measurement tool and a standalone tool for autonomous rehabilitation at home. Ubiquitous, home health or telehealth and telecare services are well explored [51–55] but remain, for the moment, at the proof-of-concept state. Algar and Valdes evaluated in their study the use of Smartphone applications as hand therapy interventions [56]. They explained how Smartphone applications could bring solutions to clinician for rehabilitation at home and how it can improve patient compliance. A first example is given for treatment of trapeziometacarpal arthrosis with two Smartphone applications which require the use of both palmar abduction and the unconscious activation of thumb muscles. Exercises including these movements are essential to increase range of motion and grip strength and to decrease pain. The second example is for treatment following distal radius fracture. Smartphone applications can provide wrist proprioceptive and joint sense exercise, whose therapeutic roles are validated for rehabilitation after wrist injuries. It now remains to assess the benefits of these applications in clinical studies involving targeted populations on rehabilitations exercises at home. Using the Smartphone for home rehabilitation exercises just started since these tools are now available to the largest number in developed countries. It was firstly studied for cardiac disease [57, 58], pulmonary rehabilitation [59], or prevention of ankles sprains [60, 61].

In their study in cardiac rehabilitation, Varnfield et al. have compared the use of a Smartphone for cardiac rehabilitation against traditional home-based rehabilitation [57]. One hundred and twenty patients with postmyocardial infarction were recruited during six months and randomly separated into two distinct groups. Uptake, adherence, and completion were evaluated. Significance for relative risk was calculated using two-sided Fisher's exact test.* chi*-square test was used for categorical variables, two-sample *t*-test was used for continuous variables, and the Wilcoxon rank-sum test was used for skewed variables. A linear mixed model regression was used to compare longitudinal changes across baseline and a preliminary multivariate analysis was used to analyze the association between nine selected baseline characteristics and outcomes. The Smartphone-based program was used with the aim of delivering exercises monitoring, motivational and educational materials via Short Text Messages (SMS) and video, and a health diary. Authors concluded that “this smartphone-based home care CR program improved post-MI CR uptake, adherence and completion.” [57], validating the use and clinical effectiveness of a Smartphone application for home care cardiac rehabilitation.

A second study, from Layton et al., aimed to determine the feasibility and the acceptability of a Smartphone-based application to monitor outpatient discharge instruction compliance in cardiac disease [58]. Sixteen patients were recruited. Smartphone was used to daily monitor medication compliance, physical activity, follow-up care, symptoms, and reading of education material. Findings suggest that stable patients used the application more than unstable patients. Acceptability was low and varied greatly but it is similar to other studies. Authors concluded that this study “demonstrated that usage alone may be a useful tool to highlight patients in need of closer monitoring” [58]. This scientific work allows validating the feasibility and acceptability of a Smartphone application for monitoring outpatient.

For prevention of ankles sprains, study from Vriend et al. led to the same acceptability results [61]. These authors have developed a Smartphone application providing an eight-week neuromuscular training program with a set of six different exercises. It was evaluated using the Reach Effectiveness Adoption Implementation Maintenance Framework. Results showed a low compliance but the app reached only 2.6% of the projected targeted population.

For their study in pulmonary rehabilitation, Marshall et al. described a model of Smartphone application which can support remote patients with chronic obstructive pulmonary disease and give them an automatic feedback during exercises [59]. This application was not yet evaluated in full patient trial.

Thus, some of these studies highlight the fact that the acceptability varied greatly [58] and, for prevention, targeted efforts have to be made to ensure that a specific population can and will be willing to use the application [61]. However, these studies confirm the feasibility and a certain acceptability to use Smartphone application for monitoring and rehabilitation at home. Following these observations, a need exists for validation studies focused on autonomous rehabilitation at home using the Smartphone as a personal physiotherapist that can bring measurement and feedback to patients to improve the follow-up between medical sessions.

## 4. Future Work

Autonomous rehabilitation could be provided by a Smartphone-based system. This system is composed of inertial sensors to measure orientations, calculation units to analyze motor control abilities, visual, auditory, and somatosensory systems to provide biofeedback to the user, screen display and headphones to provide test and/or training exercises instructions, and wireless connection to transmit data. With this system, physiotherapist could provide to patient specific and personalized exercises to optimally improve proprioceptive functions. Various proprioceptive exercises are possible: active joint repositioning training, path-of-motion training, and so forth. It was proved that, for proprioception assessment, active movements give more information from muscle and joints receptors while fatigue should be avoided [41]. Along these lines, to assess, monitor, improve, and train proprioceptive function, we have developed a specific Smartphone application called “iProprio.” “iProprio” functioning is based on the use of inertial sensors to measure active range of motion from different body part such as shoulder, elbow, or knee. The innovative part of the application is based on the fact that it proposes different active joint repositioning training with the help of different sensory feedback. All these exercises could be performed in autonomous way at home thanks to the Smartphone. The instructions can be automatically vocally or visually supplied. We are currently evaluating “iProprio” with targeted population in terms of effectiveness, efficiency, satisfaction, usability, and acceptance with a specific design model called TEMSED for “Technology, Ergonomics, Medicine, Society, Economics, and Deontology” [62].

## 5. Conclusions

In this paper, we have reported related works on clinical assessment that uses Smartphone as a joint angle measurement tool to assess proprioceptive abilities. It is mainly used for assess joint position sense and range of motion. This state of the art highlights that Smartphone applications have proved their reliability and validity for clinical uses. At this point, although their usefulness is underlined in some studies conclusions, there are no studies that have evaluated the use of a Smartphone in autonomy during home rehabilitation through exercise therapy to enhance proprioception.

## Figures and Tables

**Table 1:** Characteristics of the different studies that have been examined.

Study	Population (Sample size and age)	App	Reference	Body segment	Movement	Type	Results		
							Validity	Reliability	
								Intraobserver	Interobserver
Tousignant-Laflamme et al., 2013 [11]	Healthy volunteers (28, 23 ± 6 years)	Clinometer and Compass on iPhone	Eyeglasses with three inclinometers	Head	Flexion/extension/rotation	Active CROM	ICC between 0.50 and 0.65 but <0.50 for rotation	ICC = 0.65–0.85	ICC < 0.60

Quek et al., 2014 [12]	Healthy volunteers (21, 31 ± 9 years)	Customized Android	3D motion analyses system	Head	Flexion/extension/rotation	Active CROM	ICC = 0.53–0.98, Spearman's *ρ* = 0.52–0.98	ICC = 0.82–0.90 but poor in rotation: ICC = 0.05–0.33	N/A

Shin et al., 2012 [13]	Healthy volunteers (41, 52.7 ± 17.5 years)	Clinometer on Android	Double armed goniometer	Shoulder	Forward flexion/abduction/external and internal rotation and abduction	Passive and active ROM	LOA = 10–40° ICC(3,1) > 0.72 Pearson's correlation coefficient (PCC) = 0.79–0.97	ICC(2,1) > 0.70 except for ICC(2,1) = 0.63–0.68 (internal rotation at 90° abduction)	ICC(3,1) > 0.90

Werner et al., 2014 [14]	Healthy volunteers (24) and symptomatic patients (15)	Clinometer on iPhone	Visual estimation and standard goniometer	Shoulder	Forward flexion/abduction/external and internal rotation	Passive ROM	For healthy volunteers, ICC > 0.60 and SEM < 4.3° For symptomatic patients, ICC > 0.80 and SEM < 0.1°	N/A	For healthy volunteers, ICC > 0.60 and SEM < 10.1° For symptomatic patients, ICC > 0.60 and SEM < 5.8°

Mitchell et al., 2014 [15]	Healthy volunteers (94)	GetMyRom and DrGoniometer on iPhone	Standard goniometer	Shoulder	External rotation	Active ROM	ICC = 0.94	ICC = 0.94	ICC = 0.79

Oïhénart et al., 2012 [16]	Healthy volunteers (5)	iShould on iPhone or iPod touch	3D kinematic sensors	Shoulder	Anterior elevation and extension/abduction/adduction/internal and external rotation	Active ROM	Mean difference (%): 1.09 for RAV and 0.60 for *P* score	N/A	N/A

Johnson et al., 2015 [17]	Healthy volunteers (1)	Customized Android	Double-armed goniometer and full-scale motion capture	Shoulder	Abduction	Passive ROM	Mean difference in B&A plots = −1.7° (seated position) and 1.4° (supine position)	CCC > 0.992	CCC > 0.989

Ferriero et al., 2011 [18]	Healthy volunteers (28 pictures!)	DrGoniometer on iPhone	Plastic universal goniometer	Elbow	Elbow placed at different angles	Passive ROM	LOA = +4.51°, −5.75°	ICC = 0.998	ICC = 0.998

Ockendon and Gilbert, 2012 [19]	Healthy volunteers (5, 30–40 years)	Knee goniometer on iPhone	Telescopic-armed goniometer	Knee	Flexion	Passive ROM	LOA = 15.2° PCC = 0.947	PCC = 0.982	PCC = 0.994

Hambly et al., 2012 [20]	Healthy volunteers (96, 31 ± 11 years)	Knee goniometer on iPhone	Telescopic-armed goniometer	Knee	Flexion	Active ROM	PCC = 0.932 paired *t*-test indicates significant difference but not clinically significant	ICC = 0.894 PCC = 0.795	N/A

Jones et al., 2014 [21]	Healthy volunteers (36, 60.6 ± 6.2 years)	Simple goniometer on iPhone	Universal goniometer	Knee	Lunge forward	Active ROM	LOA = 13.1° *r* = 0.96–0.98	ICC = 0.97–0.99	N/A

Milanese et al., 2014 [22]	Healthy volunteers (6)	Knee goniometer on iPhone	Universal goniometer	Knee	Flexion	Passive ROM	CCCexpert = 0.982 CCCnovice = 0.983 CCCensemble = 0.991 CPP = −0.51 SEM < 2.7° (gonio) SEM < 1.4° (apps)	CCCexpert > 0.998 CCCnovice > 0.997	CCCexpert > 0.996 CCCnovice > 0.998 CCCtogether = 0.997

Rwakabayiza et al., 2013 [23]	Healthy volunteers (20) and symptomatic patients (20)	Knee goniometer on iPhone	Universal goniometer	Knee	Flexion/extension	Active and passive ROM	On 6 patients ICC = 0.54 (Smartphone) in active extension ICC = 0.92 (Smartphone) in active flexion (better than goniometer)	ICChealthy = 0.85 (0.75–0.94) ICCsymptomatic = 0.98 (0.96–0.99)	ICChealthy = 0.12 (0.00–0.25) ICCsymptomatic = 0.24 (0.00–0.45)

Bruyneel and Bridon, 2015 [24]	Healthy volunteers (41, 18–26 years)	Clinometer on iPhone	Hand-held bilevel inclinometer	Knee	Extension	Passive ROM	N/A	ICC > 0.76 (inclinometer) ICC > 0.72 (Smartphone) MDC < 5°	ICC > 0.64 (inclinometer) ICC > 0.64 (Smartphone) MDC < 8°

Jenny 2013 [25]	Symptomatic patients (10, 69 years)	Angle on iPhone	Navigation system	Knee	Flexion/extension	Passive ROM	LOA = 27.4° *t*-test not significant Spearman's = 0.99 B&A: good coherence	ICC = 0.81	ICC = 0.79

Ferriero et al., 2013 [26]	Healthy volunteers (1)	DrGoniometer on iPhone	Universal goniometer	Knee	Different knee angles measurement	Passive ROM	LOA = −7.5°/+10.71° B&A	ICC = 0.958	ICC = 0.994

Jenny et al., 2015 [27]	Symptomatic patients (10, 69 ± 10.8)	Goniometer Pro and DrGoniomter on iPhone	Navigation system	Knee	Flexion/extension	Passive ROM	Strong correlation and a good coherence (Levene's test, ANOVA test, Wilcoxon's test, Kendall's test, Spearman's test, and B&A). Inclinometer results differed	N/A	N/A

Andrea et al., 2014 [28]	Symptomatic patients (35)	“SmartJoint” Android and iOS application	KT 100	Knee	Lachman's test	NC	Mean ICC = 0.797 (uninvolved knee) ICC = 0.987 (involved knee)	ICC = 0.973 (uninvolved knee) ICC = 0.989 (involved knee)	ICC = 0.957 (uninvolved knee) ICC = 0.992 (involved knee)

Yoon et al., 2014 [29]	Healthy volunteers (10, 22.2 ± 1.69 years)	TiltMeter on iPhone	Digital inclinometer	Hip	Measure of femoral neck anteversion	Passive ROM	Similarly ICC	ICC(2,3) = 0.95 SEM = 1.9°–2.2°	ICC(2,3) = 0.85 SEM = 4.1°

Peters et al., 2012 [30]	Symptomatic patients (50, 67 years from 31 to 84)	Angle and Camera Protractor on iPhone	X-Rays	Hip	Measurements for total hip arthroplasty	NC	Differences are under 5% between pre- and postop	N/A	N/A

Charlton et al., 2015 [31]	Healthy volunteers (20, 23.8 ±4.6 years)	Hip ROM tester	Camera marker based 3DMA	Hip	Flexion/abduction/adduction/supine internal and external rotation	Passive ROM	ICC(2,3) > 0.88 (for 6 movements) ICC(2,3) = 0.71 (for supine external rotation)	ICC(2,3) > 0.84 (for 4 movements) ICC(2,3) = 0.63–0.68 (for 3 movements)	N/A

Vohralik et al., 2014 [32]	Healthy volunteers (20, 21–28 years)	iHandy Level on iPhone	Digital inclinometer	Ankle	Ankle dorsiflexion range	Active ROM	CPP = 0.99	ICC(2,1) = 0.97	ICC(2,1) = 0.76

Williams et al., 2013 [33]	Healthy volunteers (20, 40 ± 12 years)	TiltMeter on iPhone	Digital inclinometer	Ankle	Weight bearing lunge test	Active ROM	ICC = 0.83	ICC(2,1) = 0.81–0.85	ICC(2,2) = 0.80–0.96

Kolber et al., 2013 [34]	Healthy volunteers (30, 25.6 ± 2.1 years)	iHandy Level on iPhone	Bubble inclinometer	Spinal	Thoracolumbopelvic flexion, isolated lumbar flexion, thoracolumbopelvic extension right lateral flexion, and left lateral flexion	Active ROM	ICC(3, *k*) > 0.86 LOA = 18–30°	ICC(3, *k*) > 0.80	ICC(2, *k*) > 0.81

Izatt et al., 2012 [35]	NC (8 torso)	Scoligauge on iPhone	Scoliometer	Spinal	NC	Passive	LOA = 6.2°	ICC 95% = ±3.2°	ICC = 0.92 (absolute agreement definition) IC 95% = ±4.9°

Franko et al., 2012 [36]	NC (60 angles)	Scoligauge on iPhone	Scoliometer	Spinal	Sixty angles randomly selected	Passive	CPP = 0.99	N/A	N/A

Balg et al., 2014 [37]	Symptomatic patients (34)	Scoligauge on iPhone	Scoliometer	Spinal	Scoliosis angles	Active	ICC = 0.947 Mean difference = 0.4°	ICC = 0.961 Mean difference = 0.0°	ICC = 0.901 Mean difference = 0.1°

Qiao et al., 2014 [38]	Symptomatic patients (64, 15.7 year)	Scoligauge on iPhone	Scoliometer	Spinal	Scoliosis angles	Active	Mean angles are similar	ICC (scoliometer) = 0.954 ICC (apps) = 0.965	ICC (scoliometer) = 0.943 ICC (apps) = 0.964
